# Impact of a self-care education programme on patients with type 2 diabetes in primary care in the Basque Country

**DOI:** 10.1186/1471-2458-13-521

**Published:** 2013-05-29

**Authors:** Estibaliz Gamboa Moreno, Álvaro Sánchez Perez, Kalliopi Vrotsou, Juan Carlos Arbonies Ortiz, Emma del Campo Pena, Lourdes Ochoa de Retana Garcia, María Ángeles Rua Portu, Koldo Piñera Elorriaga, Amaya Zenarutzabeitia Pikatza, Miren Nekane Urquiza Bengoa, Rosario Sanz Echave, Tomás Méndez Sampedro, Ana Oses Portu, Lourdes Gorostidi Fano, Miren Bakarne Aguirre Sorondo, Rafael Rotaeche Del Campo

**Affiliations:** 1 Pasajes San Pedro Health Centre, Osakidetza, c/ Marinos nº 1, Pasajes San Pedro, Gipuzkoa 20110, Spain; 2 Primary Care Research Unit of Bizkaia, Osakidetza, c/ Luis Power 18 planta 4, Bilbao 48014, Spain; 3 Primary Care Research Unit of Gipuzkoa, Osakidetza, Pº Doctor Begiristain, Donostia s/n 20014, Spain; 4 Beraun Health Centre, Osakidetza, Av. Galtzaraborda 67, Renteria, Gipuzkoa, Spain; 5 Bidebieta Health Centre, Osakidetza, Pº Julio Urkijo, San Sebastián, Gipuzkoa s/n - 20016, Spain; 6 O + Berri, Fundación Vasca de Innovación e Investigación Sanitarias, Plaza Asua, 1. 48150, Sondika, Bizkaia, Spain; 7 Unidad Docente de Medicina familiar y Comunitaria de Bizkaia, Osakidetza, c/ Luis Power 18 planta 4, 48014, Bilbao, Spain; 8 Gazalbide-Txagorritxu Health Centre, Osakidetza, c/ Chile, 9-01009, Vitoria-Gasteiz, Spain; 9 Ortuella Health Center, Osakidetza, Avda. Minero, Ortuella, Bizkaia s/n - 48530, Spain; 10 Hondarribia Health Center, Osakidetza, c/ Matxin de Arzu nº 2, Hondarribia, Gipuzkoa 20280, Spain; 11 Alza Health Centre, Osakidetza, Paseo de Larratxo 95, San Sebastián, Spain

**Keywords:** Chronic disease, Diabetes Mellitus type 2, Self-management, Education, Primary care

## Abstract

**Background:**

Type 2 diabetes mellitus (DM2) is a disease with high prevalence and significant impact in terms of mortality and morbidity. The increased prevalence of the disease requires the implementation of new strategies to promote patient self-management. The Spanish Diabetes Self-Management Program (SDSMP) has proven to be effective in other settings. The objective of this study is to assess its effectiveness in terms of care for DM2 patients in primary care settings within the Basque Health Service – Osakidetza (Spain).

**Method/Design:**

This is a randomised clinical trial in which patients diagnosed with DM2, 18–79 years of age, from four health regions within the Basque Health Service will be randomised into two groups: an intervention group, who will follow the SDSMP, and a control group, who will receive usual care in accordance with the clinical guidelines for DM2 and existing regulations in our region. The intervention consists of 2,5 hour-group sessions once a week for six weeks. The sessions cover target setting and problem solving techniques, promotion of physical exercise, basic knowledge of nutrition, proper use of medication, effective communication with relatives and health professionals, and basic knowledge about DM2 and its complications. This content is complemented by educational material: books, leaflets and CDs. The primary outcome measure will be the change in glycated haemoglobin (HbA1c), and secondary outcome measures will include changes in levels of physical activity and intake of fruit and vegetables, cardiovascular risk, quality of life, self-efficacy, number of consultations and drug prescriptions. The results will be analysed 6, 12 and 24 months after the intervention.

**Discussion:**

If the intervention were to be effective, the programme should be spread to the entire diabetic population in the Basque Country and it could also be applied for other diseases.

**Trial registration:**

ClinicalTrials.gov identifier NCT01642394

## Background

Managing patients with chronic health problems represents a challenge for health services. According to the 2007 Basque Health Survey, chronic diseases are increasingly prevalent in our setting, currently, 83.5% of women and 86.1% of men aged over 65 years having some type of chronic condition [1]. In the health system, 80% of primary care consultations are related to chronic diseases, as 60% of hospital admissions and 70% of health expenditure [2].

In particular, the incidence of type 2 diabetes mellitus (DM2) has increased in our region in recent years. Indeed, it is predicted that prevalence of DM2 may reach up to 12% in the population over 30 years of age [3]. Mortality and morbidity in DM2 patients are known to be much higher than those in the general population; specifically, in diabetic patients, the mortality rate ranges from 13 to 30 deaths per 100,000 people per year, and 75% of these deaths are due to heart disease [3]. Further, it has been estimated that diabetes accounts for as much as 6.3 to 7.4% of health expenditure in our health system, while 42% of people with diabetes in our region are obese, 79.6% have high blood pressure and 22% have diabetic macroangiopathy [4]. According to the latest internal assessment data, there is room for improvement in diabetes control in our setting. Well-controlled diabetic patients, that is, those with HbA1c levels below 7%, represent just 44% of the overall diabetic population, and blood pressure is under 140/90 in only 25% of patients (unpublished data from internal assessments in the Basque Health Service, Osakidetza).

Health systems need to provide more efficient and coordinated care to patients with chronic conditions and this should be focused on decreasing rates of deterioration and improving the quality of life of patients and their families/caregivers. In relation to this, primary care has an essential role [2]. As in other developed countries, the Spanish Health System is outdated, and a new organisational model is needed to respond to the challenge of providing care to chronic patients [5]. Though several models have been proposed, the Chronic Care Model and the Kaiser Permanente model are among the most used and studied [2].

In both models, a key role is given to self-management and educational programmes, implying patient involvement in the management of their own condition. Among such programmes, the most widely used and studied structured approach is that of the Chronic Disease Self-Management Program (CDSMP) developed at Stanford University. The CDSMP is based on the self-efficacy theory of Albert Bandura [6], a social cognitive theory, which establishes two key factors in the achievement of successful behavioural change: confidence (self-efficacy) in one’s ability to perform a task, and the expectation of achieving a specific target (outcome expectation).

Despite the many systematic reviews published on the efficacy of different self-management education models [7-9], observed outcomes are heterogeneous due to the variable duration of study periods, types of interventions and target populations. What is more, programmes have not been assessed over the long-term.

In our setting, the pilot studies using this method have been promising, but have not yet been rigorously and prospectively evaluated compared to usual care [10]. A very recent trial, conducted in a different healthcare context, in male war veterans in the United States, has demonstrated that an educational intervention led by patients themselves is more effective for controlling glycaemic levels than one run by nurses [11]. Another recent study of a patient-led intervention, in primary care in Ireland, peer support found that the intervention was not effective in decreasing HbA1c levels in DM2 patients; however, the programme used was designed by the research team and placed more emphasis on social support than educational aspects [12], which is a different approach to that of the Spanish Diabetes Self-Management Program (SDSMP).

The Health Department of the Basque Country Government is promoting a new strategy for chronic patient care based on the Chronic Care Model (http://cronicidad.euskadi.net). Two of the cornerstones of this model are self-care promotion and population education. In this context, the “Active Patient” programme, based on the SDSMP which shares core elements with the CDSMP, has been proposed as a useful instrument for promoting self-care in patients with DM2. In order to assess the effectiveness of this programme, compared to usual care for DM2 patients in the Basque Health Service, we decided to conduct a clinical trial. The present paper describes the protocol we have designed for that purpose.

## Methods/design

### Primary objectives

To assess the effect of the SDSMP on glycaemic control in DM2 patients compared to usual care provided to this type of patient in primary care in the Basque Health System.

### Secondary objectives

● To assess any changes in

 o level of cardiovascular risk

 o quality of life

 o self-efficacy

 o physical activity

 o eating habits

● To evaluate the use of health resources by diabetic patients

● To assess the impact of the SDSMP on drug prescription and associated costs

● To describe the feasibility of implementing SDSMP within primary care in the Basque Health System.

### Hypothesis

An educational intervention for DM2 patients, based on the SDSMP, will be conducted by two leaders. At least one of them will be a chronic patient and have previously received specific training. This intervention will improve metabolic control, with a decrease of 0.5% or more in HbA1c levels [13], in comparison to usual health education.

### Design

A randomised clinical trial will be carried out in four health regions of the Basque Health Service (Araba, Gipuzkoa, Ezkerraldea-Enkarterri and the Bidasoa Integrated Health Organisation) involving a total of 556 DM2 patients between 18 and 79 years old, recruited proactively, as described in the section on the recruitment process below. Patients will be randomly assigned to intervention and control groups, and will be followed up over two years. Measurements will be taken at four time points: baseline, and 6, 12 and 24 months after the intervention. The research protocol has been approved by the Basque Country Clinical Research Ethics Committee (Ref. no.: 11/2010).

### Study subjects

#### Eligibility criteria

Patients must be 18 years old or above and have DM2, must be from one of four aforementioned health regions within the Basque Country, and must agree to participate in the Active Patient programme: “*Paciente Activo-Paziente Bizia*”.

#### Exclusion criteria

We will exclude individuals over 80 years of age, those with mental disorders associated with a loss of a sense of reality (schizophrenia, bipolar disorder, Alzheimer´s disease, psychosis or dementia) and those with any associated morbidity that might impede their participation in the course, such as is the case for chronic patients requiring home care.

#### Recruitment process

Eligible patients will be recruited in several ways: through advertisements for volunteers in the local media; by sending letters addressed to patients diagnosed with DM2, based on data from the Osabide electronic health record; and by their assigned health professionals (general practitioners and nurses) directly inviting them to participate.

**Figure 1:**
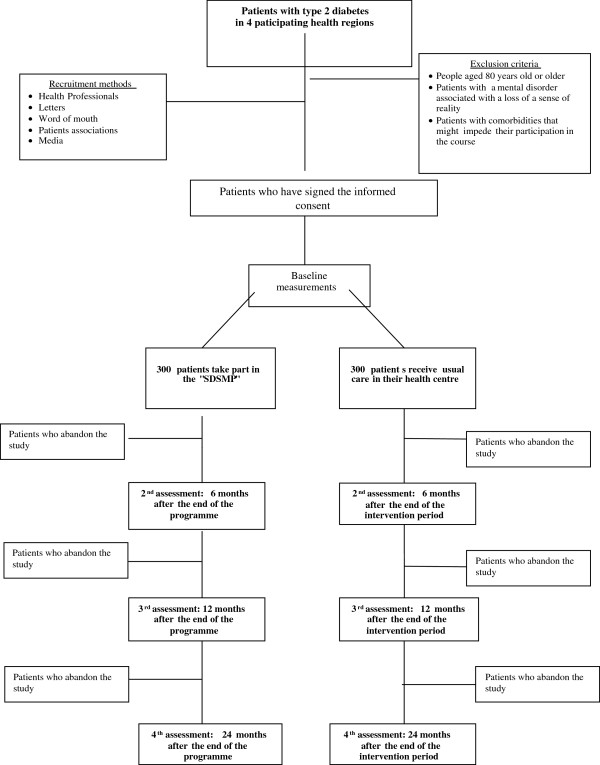
Flowdiagram for the study.

Patients will be informed about the objectives of the study, and those willing to participate will be randomly assigned to intervention or control groups, after giving written informed consent. The baseline measurements will be taken before the randomisation process (Figure 1).

#### Withdrawal from the study

Participants will be able to withdraw from the study at any point if they wish. Those in the intervention group must participate in a minimum of four educational sessions; else, they will be considered as dropouts. All those randomised will, nevertheless, be included for the statistical analysis (i.e., data will be analysed on an intention-to-treat basis).

### Randomisation

Randomisation will be performed centrally by staff other than those of the research team, using a computer-generated random number sequence and after taking the baseline measurements.

### Standardization of the intervention

#### Intervention group

The complete intervention consists of group sessions (8 to 15 people) lasting 2.5 hours, once a week for six weeks. These sessions are structured with the aim of enabling participants to acquire knowledge and skills related to the disease and its management, placing a strong emphasis on proactive tools to achieve healthier lifestyles (in terms of diet, physical exercise, management of emotions, and correct use of medication, among others).

During the sessions, patients will be taught how to set realistic achievable targets, cope with problems related to their disease, communicate more effectively with relatives and health professionals sharing their feelings, and, overall, have an active attitude towards managing their disease and towards more healthy lifestyles. The sessions are supported by educational materials: books, leaflets and CDs.

Each group will be supervised by two leaders, who have previously been trained in the SDSMP. This training consists of two parts: the first, 24-hours of theoretical training, given by two Master Trainers certified by Stanford University; and, the second, practice, which involves trainees running a workshop with patients. Having completed these two components of the training, they can be certified as leaders. At least one of the two leaders should be a chronic patient him/herself or caregiver for a chronic patient. Leaders who are themselves chronic patients, or have close personal experience of a chronic condition, show greater empathy and tend to suggest more appropriate and realistic options than health professionals. The other leader, who is a doctor or nurse, should introduce himself to patients on the courses as an SDSMP programme leader, rather than as a health professional, as this strengthens the idea of peer education.

#### Control group

Patients assigned to the control group will receive usual care for secondary prevention of DM2 in accordance with the Osakidetza primary care protocols, which are based on clinical practice guidelines [14].

### Outcome variables

Baseline measurements will be taken prior to randomisation. As noted above, the clinical data will be collected at each health centre by collaborating health professionals, previously trained by the research team. Some instruments to be used are for self-completion by patients, while others are questionnaires for use by interviewers previously trained by the research team.

Blood samples will be collected by staff at the patients’ assigned health centres, and the analysis will be performed at the reference laboratories of the four participating health regions.

#### Primary outcome measure

Change in glycosylated haemoglobin (HbA1C) levels

#### Secondary outcome measures

Changes in the following:

● Body weight, height and BMI measured in standard conditions

● Systolic and diastolic blood pressure (SBP and DBP) measured by a validated automated sphygmomanometer following the recommendations in the 2007 Osakidetza clinical practice guidelines [14]

● Total and HDL cholesterol levels

● REGICOR cardiovascular risk scores [15]

● Quality of life assessed by the Spanish version of the ADDQoL-19 [16]

● Self-efficacy assessed using the Diabetes Self-efficacy Scale from Stanford University, and the level of physical activity assessed by the 7-day Physical Activity Recall (PAR) [17] questionnaire

● Mediterranean-Dietary Quality Index (PREDIMED Study)

● Medication taken for DM and associated risk factors (hypoglycaemic, antiplatelet, antihypertensive and lipid-lowering medication)

● Daily expenditure on medication

● Use of health services (number of visits to the doctor or nurse, admissions due to diabetes-related metabolic decompensation)

#### Independent variables

Age; sex; number of years since diagnosis of diabetes; history of diabetic microvascular disease: retinopathy, nephropathy or neuropathy; history of diabetic macrovascular disease: heart disease, peripheral arterial disease or cerebrovascular accident; treatment with anti-hyperglycaemic drugs: oral hypoglycaemic agents or insulin; and preventive cardiovascular medication: antiplatelet, antihypertensive and lipid-lowering drugs.

#### Variables related to the group intervention

Leader conducting the sessions

#### Variables related to the implementation

Percentage of the overall diabetic population invited who agree to participate in the programme; percentage of patients who agree to participate that then complete the programme; number of patients who go on to become trainers for the “Active Patient” programme; results of the satisfaction survey; number and percentage of health professionals involved at each centre who conduct the regular check-ups for diabetes patients participating in the research; and number and percentage of professionals who become trainers in the Active Patient programme.

Both the intervention and control groups will have four sets of measurements taken: prior to the intervention and 6, 12 and 24 months after the intervention.

### Data management

The research team will run a training session for collaborators in the health centres, to ensure consistency in the data collection. In addition, printed and electronic support material will be available in each participating centre.

The researchers at each centre will collect the data using a form designed for this purpose. These data will be then stored in a password-protected Access database using double-data entry designed for the purpose. Similarly, the interviewers will enter the data into an Access database designed for the purpose. A report on the quality of data obtained will be produced once a month.

### Sample size

Based on the results of a pilot study with 171 patients, we found a standard deviation (SD) of 1.2% in HbA1c and, given that that the mean SD in literature is around 2%, we used an SD of 1.5% to calculate the sample size. Considering an intra-class correlation of 0.05 [18] and an average of 10 participants per group, we calculated that we needed 277 participants per study arm to detect a clinically relevant difference of 0.5% in HbA1c levels at 12 months, with 80% power, with p-value of 0.05 and assuming a dropout rate of 15%.

### Data analysis

The categorical variables will be presented as frequencies and percentages (%) while the continuous variables will be reported as means and SDs. Patients in the intervention and control groups will be compared on an intention-to-treat basis. To assess the intervention effect, we will compare changes observed in each of the groups using Chi-square tests for categorical variables, and Student’s t-tests for continuous variables in the two independent groups, for each of the repeated measurements. To adjust variables that may confound or moderate the effect of the intervention, we will perform stratified analysis and multivariate regression analysis. Multilevel analysis, i.e., generalized mixed-effects models, will be used to estimate baseline and multivariate-adjusted between-group mean differences, adjusted odds ratios (AORs), and 95% confidence intervals (CIs) at the patient level, taking into account the clustered structure of data for patients for whom the intervention has been led by different individuals.

Longitudinal generalized mixed models will be used to analyse the overall trend in changes in the outcome measures taking into account the repeated measurements for each patient and also the clustered structure of data. These models will be linear for continuous variables, and logistic for categorical variables. Time will be considered in these models as a continuous variable, based on linear, quadratic or logarithmic functions, or as a categorical variable, with several correlated measurements for each individual. The least restrictive option with the best fit to the data will be chosen. The intervention, the time of measurement and intervention-time interaction will be included as fixed effects in the models. Patients and leaders will be included as random effects on the intercept and on the time slope. These models will be extended to include variables that may confound or modify the effect of the intervention, this being guided by the stratified analysis. At this stage, results will be presented as coefficients with their respective 95% confidence intervals. The analysis will be performed using SAS v. 9.2, SPSS v. 19 and R.

## Discussion

The objective of this clinical trial is to assess the effectiveness of the SDSMP in contrast to usual care for DM2 patients within primary care in the Basque Country. This intervention, while providing knowledge concerning the disease and its management, is different from the usual health educational models in the sense that it involves peer group education and puts considerable emphasis on reinforcing attitudes and skills that enhance proactivity, such as target setting, problem solving, positive thinking, communication techniques, dealing with feelings, and proper use of prescribed treatments. This should empower patients to more actively manage their lifestyles, changing towards healthier habits and achieving improvements in diet, physical exercise, correct use of medications, and relationships with their healthcare provider, as well as with their relatives and friends.

Existing self-care education programmes [7] are based on a range of different methods and measure different variables and, further, they have not been assessed by prospective and controlled studies in our health system. On the other hand, these types of intervention are often mentioned in debates about the changes required in health systems to face the current demographic situation. It is, therefore, necessary to provide good quality evidence to guide the selection of appropriate interventions by undertaking controlled randomised clinical trials assessing their effectiveness [19].

In our health setting, the feasibility of implementing such a programme would rely on it being integrated into the everyday practice of health professionals and on gaining support and acceptance among patients as well as leaders. If this research in our setting yields the hoped-for results, this would give us a tool for improving the care provided to patients with diabetes and other chronic diseases. The courses could be adapted to different disorders and thereby improve self-care among a wider population, leading to more self-efficacy, greater quality of life, better clinical outcomes and proper use of healthcare services, and therefore a more efficient use of resources.

### Limitations of the study

The selection criteria may limit the external validity of the intervention as otherwise eligible patients with advanced age and comorbidity will be excluded. Further, it is necessary to recognise that participation in any training activity implies a degree of willingness on the part of the patient. We consider that this predisposition should not represent a bias in the study, given that performing the randomisation after patients have shown willing to participate should ensure comparability between groups. On the other hand, working patients, in particular, may have constraints on attending group sessions due to other commitments. To limit the impact of this problem, the courses will be offered at as wide a range of times as possible. The clinical workload of the health professionals involved in the research may, however, impose restrictions on the timetable of the training sessions.

## Competing interests

The authors have no competing interests to declare.

## Authors’ contributions

EG, JCA, RR, KP and AS have participated in the conception and design of the study as well as in the planning, analysis and writing of the protocol. All members of the Active Patient Research Group participated in the design of the study and revising the draft critically for important intellectual content and all authors have given final approval of the version submitted for publication.

## Authors’ information

EG: Principal investigator; Member of the Kronikgune research group on active and healthy ageing; Head of the Osakidetza Active Patient Programme; and Member of the Action Group A1 of the European Innovation Partnership on Active and Healthy Ageing.

AS: Member of the Spanish Research network for preventive activities and health promotion (redIAPP).

JCAO: Family doctor; and teaching representative in the Communication and Health programme of the Spanish Society of Family and Community Medicine (semFYC).

AZP: Family doctor; coordinator of the family care group of the semFYC and Basque Society of Family and Community Medicine (OSATZEN); as well as member of the OSATZEN Communication and Health and Fibromyalgia groups.

RRC: Member of the Kronikgune research group on knowledge management and coordinator of the Evidence-based Medicine Group of the semFYC.

All the authors, except AS, are members of the Osakidetza Active Patient working and research groups.

Kalliopi Vrotsou, Estibaliz Gamboa Moreno, Juan Carlos Arbonies Ortiz, Emma del Campo Pena, Lourdes Ochoa de Retana Garcia and Rafael Rotaeche Del Campo Primary Care Research Unit of Gipuzkoa.

Álvaro Sánchez Perez Primary Care Research Unit of Bizkaia.

## Pre-publication history

The pre-publication history for this paper can be accessed here:

http://www.biomedcentral.com/1471-2458/13/521/prepub
